# Myocardial function using two dimension speckle-tracking echocardiography in children with celiac disease

**DOI:** 10.1007/s00431-023-05343-z

**Published:** 2023-12-07

**Authors:** Doaa El Amrousy, Walid Elshehaby, Radwa Elsharaby, Shaimaa Badr, Mohamed Hamza, Amany Elbarky

**Affiliations:** 1 Pediatrics Department, Faculty of Medicine, Tanta University, Tanta, Egypt; 2 Clinical Pathology Department, Faculty of Medicine, Tanta University, Tanta, Egypt

**Keywords:** Celiac Disease, 2D-STE, Echocardiography, Cardiac function, Tissue Doppler

## Abstract

The prevalence of cardiac complications linked to celiac disease (CD) is on expanding. This study aimed to evaluate the cardiac function in children with CD using two dimensional speckle tracking echocardiography (2D-STE) to detect early myocardial dysfunction, if any. This cross-sectional study included 40 children with CD as the patient group and 40 healthy age- and sex-matched children served as the control group. High sensitive troponin T (Hs-troponin T), anti-tissue transglutaminase immunoglobulin A (tTG-IgA), hemoglobin, ferritin, albumin, and vitamin D levels were measured in all participants. Conventional, tissue Doppler imaging (TDI), and 2D-STE were performed for all included children. Conventional echocardiographic parameters showed no significant difference between the two groups. Left ventricular global longitudinal strain (LV GLS) obtained by 2D-STE was substantially lower in children with CD than the control group; however, myocardial performance index (MPI) obtained by TDI was significantly higher in children with CD. Hs-troponin T levels were comparable in both groups. LV GLS was positively correlated with hemoglobin, ferritin, and albumin level, but it was inversely correlated with the duration of the disease and anti tTG-IgA.

*    Conclusion*: 2D-STE can detect subclinical early cardiac dysfunction in children with CD and this cardiac injury correlated to the duration and severity of the disease and some nutritional deficiency in these children.

**Table:** 

**What is Known:** *• The prevalence of cardiac complications linked to celiac disease (CD) is on expanding*. *• Only one study evaluated cardiac function in children with CD using two dimensional speckle tracking echocardiography (2D-STE)*.	
**What is New:** *• Our study found that 2D-STE can detect early subclinical cardiac dysfunction in children with CD. Cardiac injury in theses children correlated to the duration and severity of the disease, hemglobin, ferritin, and albumin levels*.

**What is Known:**

*• The prevalence of cardiac complications linked to celiac disease (CD) is on expanding*.

*• Only one study evaluated cardiac function in children with CD using two dimensional speckle tracking echocardiography (2D-STE)*.

**What is New:**

*• Our study found that 2D-STE can detect early subclinical cardiac dysfunction in children with CD. Cardiac injury in theses children correlated to the duration and severity of the disease, hemglobin, ferritin, and albumin levels*.

## Introduction

Consumption of gluten and associated prolamins in genetically susceptible individuals can result in celiac disease (CD), an immune-mediated systemic illness. The clinical characteristics of CD might vary considerably. A variety of clinical signs, including chronic diarrhea, malabsorption, weight loss, and nutritional deficits might be present in addition to asymptomatic variants [1]. Extra-intestinal symptoms of CD such as anemia, osteopenia, short stature, neurological abnormalities, and cardiovascular disorders are frequently present [2].

Cardiovascular disorders linked to CD are becoming a growing source of concern [3]. Among the comorbidities of celiac disease, autoimmune myocarditis and idiopathic dilated cardiomyopathy are well-known to cause significant morbidity and mortality [4]. Many postulations have been madeto explain how cardiomyopathy develops. According to one notion, dietary deficiencies caused by intestinal malabsorption can cause cardiomyopathy [5].

According to a different hypothesis, abnormal intestinal absorption increases the number of antigens and infectious agents that are absorbed, which in turn triggers the immune system and damages the myocardium and small intestine directly through the immune response [6]. However, it is debatable whether a gluten-free diet can stop the advancement of cardiac affection [7].

Few studies acknowledged that tissue Doppler echocardiography (TDE) may help to identify subclinical ventricular dysfunctions in children with CD [8, 9]. Two-dimensional speckle tracking echocardiography (2D-STE) is a new echocardiographic modality that can measure myocardial strain and assess deformation by tracking displacement of acoustic markers in the myocardium which is an emerging tool to characterize cardiac function. It has been proven to be more sensitive and more specific to assess early subclinical myocardial dysfunction compared to conventional echocardiography in pediatrics [10, 11]. There is only one study that used 2D-STE to assess subclinical cardiac dysfunction in children with CD [12]. However, the association between cardiac injury and various laboratory investigations in these children has not been fully investigated. To the best our knowledge, no previous study has reported the association between 2D-STE and various laboratory investigations in children with CD.

In this study, we aimed to evaluate cardiac function and identify any subclinical myocardial dysfunction in children with CD using 2D-STE and to correlate 2D-STE with various laboratory investigations in these children.

## Methods

This cross-sectional study was carried out between December 2021 and December 2022 on 40 children with CD who were recruited from those attending gastroenterology outpatient clinic, Pediatrics Department, Tanta University Hospitals as the patient group. Forty healthy children of matched age and sex served as the control group. Informed consent was obtained from all patients’ parents. This study was approved by the Ethics Committee of our Faculty of Medicine.

Children were diagnosed with CD according to the criteria of the European Society for Paediatric Gastroenterology, Hepatology, and Nutrition (ESPGHAN) [13]. All our patients were on gluten-free diet.

Children with systemic disorders that influence cardiac functions such as hypertension, diabetes mellitus, or thyroid dysfunction, those who were taking medications that could affect cardiac function or had pre-existing cardiac conditions were excluded from the study.

All children were subjected to the following:

(I) *Medical history review*: All participants provided a complete medical history which included their demographic information, past medical history, age at diagnosis, and dietary history to evaluate dietary compliance to gluten-free diet.
(II) *Physical examination*: The physical examination included a general condition assessment, local system examination, and vital signs recording. A special focus was placed on the assessment of any indications of specific micronutrient deficiency through examination of the skin, hair, and nails. Anthropometric measurements were acquired, including weight, height, and body mass index (BMI). The weight was recorded using a Granzia digital weighing scale (PSTG-80, Italy), while the height was determined using a Germany anthropometer (Seca 216). The BMI was calculated as kg/m^2^. The National Centre for Health Statistics’ matching growth charts were used to plot all the measurements and to calculate *Z* scores [14].
(III) *Routine investigations*: Complete blood count, serum ferritin, C-reactive protein, liver and renal function tests, serum lipid profile, serum albumin, and serum 25 OH vitamin D were measured.
(IV) *Anti-tissue transglutaminase antibody (Ttg-IgA)*: was measured using enzyme linked immunosorbent assay (ELISA).
(V) *Highly sensitive troponin T (Hs-troponin T)*: Immediately after the specimen was taken, 5 ml was randomly collected and extracted. Samples were stored at 20 °C. The supernatant was collected after centrifuging serum samples obtained by coagglutination at room temperature for 10–20 min for 20 min at a speed of 2000–3000 rpm. Centrifugation was repeated if precipitate was discovered. The kit uses a double-antibody sandwich (ELISA) [15]. It was measured by ng/ml.
(VI) *Echocardiography*: Full echocardiography examination with an experienced pediatric cardiologist was carried out for all included children using (Vivid 9; GE Healthcare, Horten, Norway) with 3.5 MHz, S7 and V3 matrix real-time three-dimensional probes, to assess cardiac function. Digital loops were stored on the hard disc of the echocardiography machine and transferred to a workstation (Echo PAC PC, 113; GE Healthcare) for offline analysis.

*Conventional echocardiography and Doppler examinations* were performed to measure left ventricular fraction shortening (LV FS), LV ejection fraction (LV EF), LV end-diastolic diameter (LVEDD), LV end-systolic diameter (LVESD), interventricular septal thickness, peak early diastolic filling velocity (E wave), peak late diastolic velocity (A wave), and early to late diastolic transmitral flow ratio (E/A). Where LV FS = (LVEDD − LVESD/LVEDD) × 100 and LV EF = (LVEDD^3^ − LVESD^3^/LVEDD^3^) × 100.

*Tissue Doppler imaging (TDI)* was used to assess the systolic myocardial velocities at the basal segments of the lateral, septal, and anterior walls (S) as well as the LV diastolic function by measuring the early and late diastolic myocardial velocities and their ratios (*E′*, *A′*, and *E′*/*A′*, respectively). The myocardial performance index (MPI) of LV was also measured by dividing the isovolumic time by the ejection time. We adjusted frame rate between 100 and 150 frames/s for obtaining the optimal myocardial tissue images.

### Two-dimensional speckle tracking echocardiography

First, select images of the apical 4-chambers, the apical 2-chambers, and the apical 3-chambers views, and then we enter the 2D-strain mode under the *Q*-analysis, respectively, and manually trace the left ventricular endocardium border at the end of systole. After that we adjusted the endocardial envelope winding accurately, and ensure the region of interest (ROI) contains complete ventricular wall information. Epicardial tracking was performed by the computer automatically. Tracking can be adjusted manually to increase tracking quality if needed. Finally, click the approve key to confirm the tracking. The software calculates the left ventricular global longitudinal strain (LV GLS) automatically ^(11)^. We adjusted frame rate between 60 and 90 to capture the optimal myocardial tissue definition. For all measures, the mean of three consecutive cardiac cycles was taken.

### Assessment of reproducibility

Echocardiographic examinations were repeated by second experienced pediatric cardiologists who were blinded to the study groups and to the reports of the first echocardiographers to measure the inter-observer reliability. Twenty randomly selected echocardiography examinations were repeated 1 week later by the same operator to assess the intra-observer reliability using intraclass correlation (ICC).

Laboratory investigations as well as echocardiographic examinations were performed at the same day. The primary outcome of this study was to assess early myocardial dysfunction in children with CD using 2D-STE. The secondary outcome was to correlate LV GLS with various clinical, laboratory, and echocardiographic data in these patients.

### Statistical analysis

Statistical analysis was performed using SPSS v26 (IBM Inc., Armonk, NY, USA). The mean and standard deviation (SD) were used for quantitative data if normally distributed, while frequency and percentage were used to display qualitative data. Shapiro-Wilk test was used to assess normality of the data. Unpaired Student’s *t*-test was employed to compare the mean between the two groups, while Fisher’s exact test or chi-square test were used to compare qualitative data. Pearson correlation coefficient was used to assess correlation between LV GLS and various clinical, laboratory, and echocardiographic data in children with CD. Statistical significance was set at *P* < 0.05.

## Results

Forty children with CD and 40 healthy controls were enrolled in the study. The demographic data of the studied groups and their anthropometric measurements are shown in Table 1. In comparison to healthy controls, children with CD had significantly lower weight, height, and BMI *Z* scores. The mean duration of disease in patients was 16.2 ± 7.5 months. Moreover, children with CD had significantly lower levels of hemoglobin, ferritin, albumin, serum 25 OH vitamin D, and TG-IgA compared to the control group, whereas Hs-troponin T levels were comparable in both groups (Table 1). Dietary compliance rate of patients with CD was 80%.

**Table 1:** The Demographic data, anthropometric measurements, and laboratory investigations of the studied groups

Parameters	CD group (*N* = 40)	Control group (*N* = 40)	*P* value
Age (years)	8.5 ± 2.4	8.9 ± 2.6	NS
Sex (male:female)	16:24	19:21	NS
Duration of illness (months)	16.2 ± 7.5	--	--
Weight *Z* Score	− 1.4 ± 0.8	0.4 ± 0.2	< 0.001
Height *Z* Score	− 2 ± 1.1	0.3 ± 0.1	< 0.001
BMI (Kg/m^2^)	16.2 ± 3.8	17.4 ± 1.5	NS
BMI *Z* Score	− 0.8 ± 1.1	0.3 ± 0.6	0.04
Hb (g/dl)	8.7 ± 1.1	12.3 ± 0.9	< 0.001
Serum ferritin (ng/mL)	15.1 ± 4.3	80.5 ± 18.2	< 0.001
Serum albumin (g/dl)	3.3 ± 0.5	4.5 ± 0.3	0.001
Serum vit D (ng/ml)	21.3 ± 4.5	30.1 ± 5.1	0.001
tTG-IgA (U/ml)	15.3 ± 2.1	0.9 ± 0.2	< 0.001
Hs-troponin T	0 (0.00%)	0 (0.00%)	1
	40 (100.0%)	40 (100.0%)	

*NS* non-significant, *BMI* body mass index, *Hb* hemoglobin, *HS* highly sensitive, *SD* standard deviation, *anti tTG-IgA* anti-tissue transglutaminase immunoglobulin A

Abdominal distention (35%) and abnormal bowel habits (45%) were the two most common abdominal complaints among our patients. The most notable extra-intestinal signs were failure to gain weight (80%), refractory iron deficiency anemia (70%), and short stature (65%). Additionally, (7.5%) of patients had angular stomatitis, (15%) of patients had nail white spots, and (20%) of patients suffered hair loss.

**Fig. 1:**
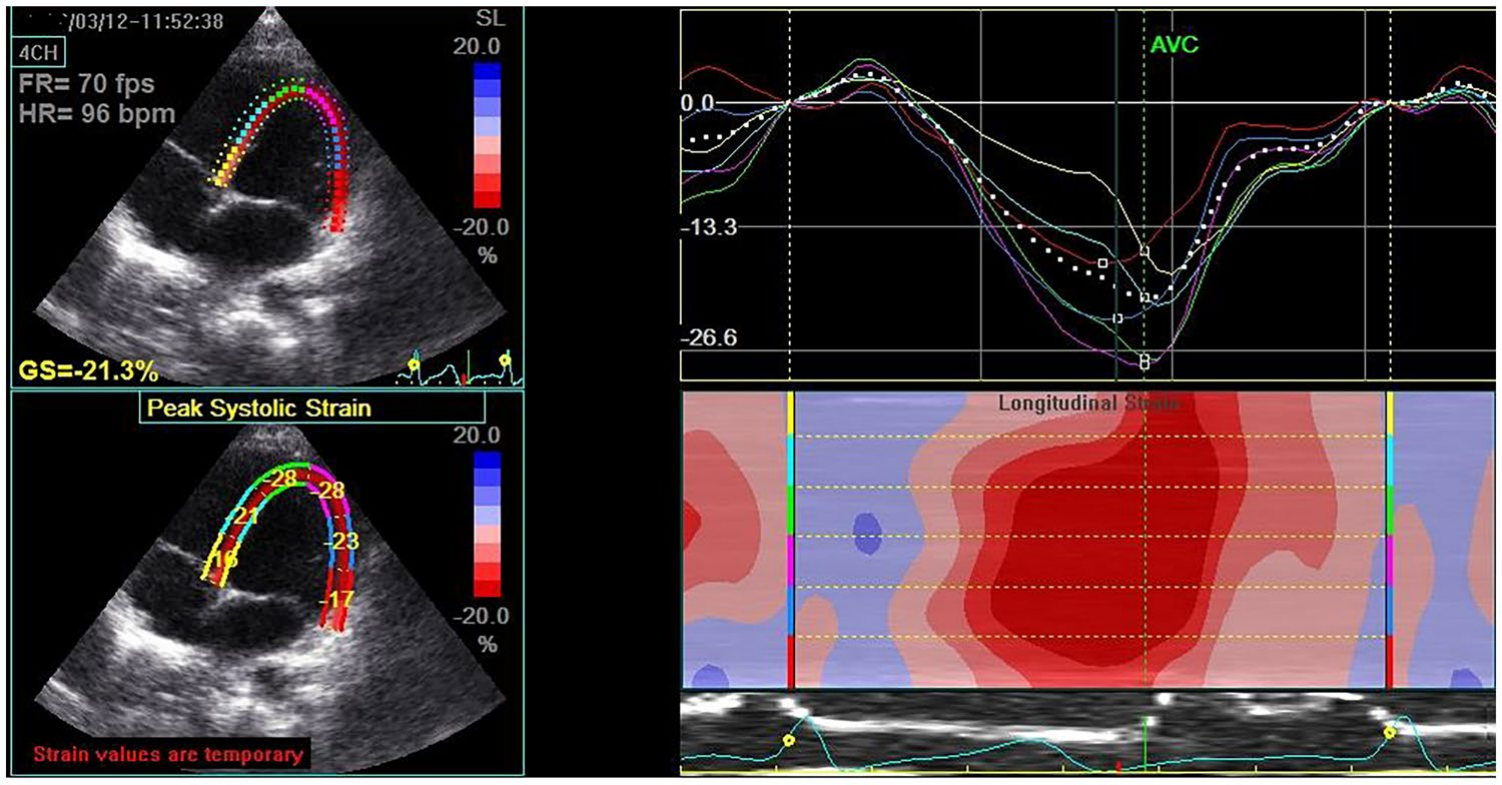
2D-GLS in a healthy control where LV GLS = -21.3%

**Fig. 2:**
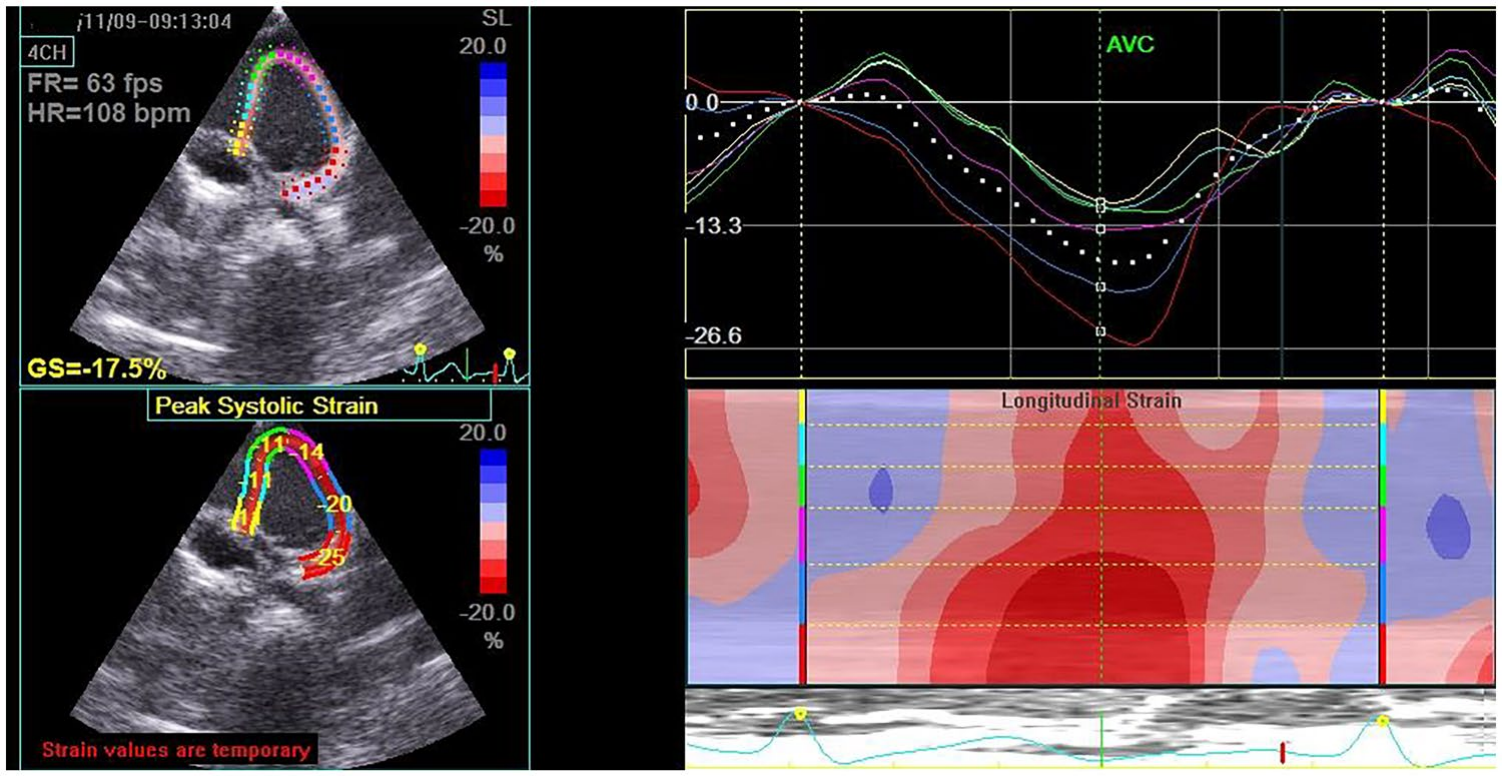
2D-GLS in a child with CD where LV GLS =  − 17.5%

Regarding conventional echocardiographic measures, there was no significant different between the two groups as regards LVEED, LVESD, septal thickness, LV EF%, LV FS%, or mitral E/A. Moreover, mitral annulus velocity (S) and mitral *E′*/*A′* obtained by TDI were comparable in both groups. However, children with CD had a significantly higher MPI compared to the control group. Interestingly, 2D-STE showed that children with CD had a significantly lower 2D-GLS compared to the control group (*P* < 0.006) (Table 2). Figures 1 and 2 show 2D-GLS in a normal control and in a child with CD, respectively.

**Table 2:** Echocardiographic parameters in the studied groups

Parameters		CD (*N* = 40)	Control (*N* = 40)	*P* value
LVEDD (cm)		3.5 ± 0.4	4.1 ± 0.7	NS
LVEDD *Z* score		1.1 ± 0.6	1.2 ± 0.4	NS
LVESD (cm)		2.8 ± 0.3	3 ± 0.5	NS
LVESD *Z* score		1 ± 0.5	1.2 ± 0.3	NS
Septal thickness (cm)		0.81 ± 0.2	0.86 ± 0.3	NS
LV EF%		69 ± 8.1	72.3 ± 6.9	NS
LV FS%		32.8 ± 2.4	35.6 ± 6.5	NS
Mitral E/A		1.1 ± 0.2	1.3 ± 0.1	NS
TDI	LV S	5.7 ± 0.9	6.3 ± 0.1	NS
	*E′*	11.6 ± 0.8	12.3 ± 0.9	NS
	*A′*	7.8 ± 0.4	7.1 ± 0.6	NS
	Mitral *E′*/*A′*	1.6 ± 0.3	1.8 ± 0.3	NS
	LV MPI	0.5 ± 0.1	0.4 ± 0.09	0.03
2D-STE	LV GLS	17.3 ± 4.2	20.1 ± 1.3	0.006

*NS* non-significant, *LVEDD* left ventricular end-diastolic diameter, *LVESD* left ventricular end-systolic diameter, *EF* ejection fraction, *FS* fractional shortening, *E/A* early to late diastolic transmitral flow velocity, *TDI* tissue Doppler imaging, *S* peak velocity during ventricular systole, *A′* peak velocity during atrial contraction, *E′* peak velocity during early ventricular diastole, *MPI* myocardial performance index, *2D-STE* two-dimensional speckle tracking echocardiography, *LV GLS* left ventricular global longitudinal strain

There was a significant positive correlation between LV GLS and hemoglobin level, serum ferritin, and albumin level. However, there was a significant negative correlation between LV GLS and both duration of illness and anti tTG-IgA levels. There was no significant correlation between LV GLS and weight, height, BMI, vitamin D level, or LV MPI (Table 3).

**Table 3:** Correlation between LV GLS and various clinical, laboratory, and echocardiographic parameters

Parameters	LV GLS	
	*r*	*P*
Weight	0.23	NS
Height	0.31	NS
BMI	0.36	NS
Duration of illness	− 0.45	0.02
Hb	0.42	0.04
Serum ferritin	0.5	0.03
Serum vit D	0.29	NS
Albumin	0.62	0.01
Anti tTG-IgA	− 0.83	0.001
LV MPI	− 0.54	NS

*BMI* body mass index, *Hb* hemoglobin, *anti tTG-IgA* anti tissue transglutaminase immunoglobulin A, *LV MPI* left ventricular myocardial performance index, *LV GLS* left ventricular global longitudinal strain

The inter- and intra-observer reliability was excellent for LV GLS obtained by 2D-STE and showed ICC of ˃ 85%.

## Discussion

CD has been linked to an increased risk of cardiovascular morbidity and mortality [16]. The autoimmune process, which is the most plausible theory, is one of the proposed mechanisms for the development of cardiac affection in celiac patients, along with nutritional deficiencies that result from chronic malabsorption, myocardial damage brought on by the absorption of various infectious agents or luminal antigens due to changes in the permeability of the intestine, and other mechanisms [5]. To identify early subclinical myocardial dysfunction, the current study attempted to evaluate the impact of CD on cardiac function using 2D STE.

In the current study, there was no statistically significant difference between the two groups using the conventional echocardiographic parameters. This agreed with the result of other investigators who studied the impact of CD on myocardial function using conventional echocardiography and TDI and they found no evidence of a statistically significant difference between cardiac measurements in both cases and controls using conventional echocardiography [17–19].

In our study, MPI (which is a sensitive measure of systolic and diastolic functions of the ventricles) was significantly higher in children with CD compared to the control group. Similar findings were reported by other investigators [17, 20, 21].

Our results showed that LV GLS was significantly lower in children with CD compared to the control group. This agreed with the results obtained by Deveci et al. who reported that 2D STE is superior to conventional and TDI echocardiography for assessing subclinical carditis in children with CD [12].

Hemodynamic factors affect the measurements of LVEF. Image quality, understanding of LV geometry, and operator’s experience are all necessary for accurate assessment of conventional LVEF. The LVEF’s limited sensitivity makes it difficult to identify subtle alterations, which further hinders the early identification of cardiac dysfunction. Therefore, a decline in the LVEF indicates a somewhat advanced level of deterioration of the LV systolic function. The recent method of 2D-STE was reported to be the most sensitive echocardiographic method for identifying the subclinical cardiac dysfunctions in several diseases as it is operator and Doppler angle independent and easy to measure [10]. SD-STE may identify minute alterations in cardiac functions across a broad range of cardiac conditions at an early stage that help in the change of treatment strategy to prevent overt cardiac dysfunction. The earliest cardiac mechanics be affected is the longitudinal strain due to the early affection of subendocardial region. Thus, it is the gold standard strain for early detection of cardiac dysfunction in several diseases [10–12]. Early detection of cardiac dysfunction in these children is of great importance as it helps to identify risk stratification and to change the strategy of treatment in these children in order to control the decrease to prevent the occurrence of overt cardiac dysfunction.

There are several theories explaining the link of CD and myocardial disease. Nutritional deficiency due to malabsorption may play a role. Increased intestinal permeability of luminal antigen that can damage the heart is another mechanism. The most plausible theory is that both illnesses could be caused by autoimmune processes [22].

Interestingly, albumin level in children with CD was significantly lower in children with CD compared to the control group. Furthermore, there was a significant positive correlation between LV GLS and albumin level in children with CD. Albumin level is expected to be low in children with CD due to decreased absorption and increased protein leakage due to loss of villi [23]. The potential role of decreased albumin levels in cardiac dysfunction could be due to its anti-oxidant and anti-inflammatory and oxidative stress and inflammation are important pathways to the development of cardiac dysfunction [24, 25]. Albumin has a strong antioxidant activity which is due to the abundant sulfhydryl groups that allows scavenging of free radicals [24], and also due to albumin’s binding properties to free metal ions such as copper and iron decreasing their availability to react with other molecules to generate free radicals [26]. Moreover, albumin exerts an indirect antioxidant activity through binding to bilirubin thus inhibiting lipid peroxidation [27]. There is also convincing evidence that hypoalbuminemia independently predicts incident heart failure [28]. Furthermore, albumin is considered a negative acute phase reactant and serum levels decrease during inflammatory states, hence the more severe inflammatory states in children with CD, the lower serum albumin levels [29].

Our results showed that hemoglobin and serum ferritin levels were significantly lower in children with CD and they were positively correlated with LV GLS. Anemia is common in children with CD due to malabsorption of iron and vitamins besides the inflammatory state of the disease that leads to anemia of chronic disease [30]. It is reported that iron deficiency anemia had a deleterious effect on the heart up to cardiomyopathy and heart failure due to cardiac tissue hypoxia that leads to myocyte dysfunction besides increased sympathetic nervous activity that affect the cardiac function [31].

In our study, anti-tissue transglutaminase antibody was significantly increased in patients with CD and was inversely correlated with LV GLS. This agreed with the results of Deveci et al. [12] who reported that patients with CD who had positive anti-tissue transglutaminase antibody tests had considerably lower longitudinal and radial left ventricular strain than the control group. Anti tTG-IgA is considered one of the important diagnostic markers of CD by ESPGHAN [13]. High anti tTG-IgA is correlated with the severity of mucosal damage which explained its inverse association with cardiac function in children with CD. Also, it may support the hypothesis of autoimmune theory of cardiac injury in these patients [32].

Interestingly, our study reported that there is a subclinical cardiac dysfunction in children with CD and this injury was correlated to the duration and severity of the disease and some nutritional deficiency namely iron and albumin deficiency. Hence, the importance of periodic follow up of these patients with 2D-STE, the correction of nutritional deficiency, and the optimal treatment of CD for early detection, risk stratification, and prevention of this cardiac dysfunction.

The study’s main limitations were the small number of participants, the lack of long-term follow-up, lack of nutritional assessment of the patients, and lack of multivariate analysis to tease out the important predictors.

Strength of our work: besides confirming the results of cardiac affection in children with CD using 2D-STE in spite of normal conventional echocardiographic examinations, we stressed on the correlation of various laboratory investigations with 2D-GLS revealing that cardiac injury was related to the duration and severity of the disease and some nutritional deficiency in these children.

## Conclusions

2D-STE can detect early subclinical cardiac dysfunction in children with CD and this cardiac injury correlated to the duration and severity of the disease and some nutritional deficiency in these children. Hence, we recommend that 2D-STE can be used as screening echocardiography modality in routine follow up of children with CD.

## Data Availability

All required data are available from the corresponding author on reasonable request.
